# Tracing the Origin, Spread, and Molecular Evolution of Dengue Type 1 Cases That Occurred in Northern Italy in 2023

**DOI:** 10.3390/pathogens13121124

**Published:** 2024-12-19

**Authors:** Greta Romano, Guglielmo Ferrari, Antonino Maria Guglielmo Pitrolo, Francesca Rovida, Antonio Piralla, Fausto Baldanti

**Affiliations:** 1 Microbiology and Virology Department, Fondazione IRCCS Policlinico San Matteo, 27100 Pavia, Italy; greta.romano01@universitadipavia.it (G.R.); guglielmo.ferrari01@universitadipavia.it (G.F.); antoninomariag.pitrolo01@universitadipavia.it (A.M.G.P.); f.rovida@smatteo.pv.it (F.R.); f.baldanti@smatteo.pv.it (F.B.); 2 Department of Clinical, Surgical, Diagnostic and Paediatric Sciences, University of Pavia, 27100 Pavia, Italy

**Keywords:** dengue virus, outbreaks, metagenomics, phylogenetics, phylodynamics, phylogeography

## Abstract

The dengue virus (DENV) is a mosquito-borne flavivirus endemic to many tropical and subtropical regions. Over the past few decades, the global incidence of dengue has risen dramatically, with the virus now present in over 100 countries, putting nearly half of the world’s population at risk. This increase is attributed to several factors, including urbanization, climate change, and global travel, which facilitate the spread of both the virus and its mosquito vectors. While dengue is primarily associated with tropical regions, outbreaks in temperate areas are becoming increasingly common due to the spread of *Aedes albopictus*, a competent vector for DENV that can adapt to cooler climates. This study investigates the molecular dynamics and geographic evolution of DENV type 1 (DENV-1) strains isolated from 13 patients during an autochthonous outbreak in Lombardy, Northern Italy, between August and September 2023. Additionally, *Aedes albopictus* mosquitoes were collected from a neighboring area to assess their potential role in the outbreak. A metagenomic approach was used to recover DENV-1 consensus sequences from clinical samples. Genotype classification and phylogenetic analyses were performed using Bayesian methods and a comprehensive dataset of DENV-1 sequences from other countries. The Italian autochthonous strains clustered with South American strains collected between 2020 and 2023, specifically those belonging to genotype V, subtype D. Bayesian analysis estimated a mean evolutionary substitution rate of 8.234 × 10^−4^ substitutions per site per year (95% HPD interval: 7.1448 × 10^−4^–9.3343 × 10^−4^), with the time to the most recent common ancestor (tMRCA) dating back to 1972 (95% HPD interval: 1968–1976). These findings suggest the likely introduction of the virus into the region from endemic areas in South America, followed by local transmission. This study offers valuable insights into the dynamics of the DENV-1 outbreak in Lombardy, underscoring the importance of genomic surveillance in monitoring viral spread and evolution. The findings emphasize the critical need for enhanced molecular and entomological surveillance to detect and respond to emerging autochthonous DENV cases in temperate regions where competent vectors, such as *Aedes albopictus*, are present. Public health strategies should prioritize integrated vector management, real-time genomic monitoring, and awareness campaigns to mitigate the risk of future outbreaks. These measures are essential to address the growing threat posed by the geographic expansion of the dengue virus.

## 1. Introduction

Dengue virus (DENV) is a mosquito-borne, single-stranded, positive-sense RNA flavivirus. It is endemic in many tropical and subtropical regions, particularly in the Americas, where its primary transmission vectors, *Aedes aegypti* and *Aedes albopictus* mosquitoes, are prevalent. However, over the past 20 years, the *Aedes albopictus* mosquito has rapidly expanded its range into temperate climates and is now widespread across significant parts of southern Europe and the United States [1].

DENV infection presents with variable clinical manifestations, ranging from asymptomatic cases to severe, life-threatening forms such as dengue hemorrhagic fever. Symptoms can include the sudden onset of fever, arthralgia, myalgia, retro-orbital headaches, maculopapular rash, leukopenia, vascular leakage, and, in rare cases, encephalitis [1]. The DENV genome is approximately 11 kilobases long and contains an open reading frame (ORF) that encodes ten mature proteins. These include three structural proteins—capsid (C), pre-membrane/membrane (prM/M), and envelope (E)—and seven nonstructural (NS) proteins: NS1, NS2A, NS2B, NS3, NS4A, NS4B, and NS5. Among these, the E glycoprotein is the most immunologically significant; however, other proteins also play crucial roles in viral replication [2].

Dengue disease is caused by four antigenically distinct serotypes (DENV-1 to DENV-4), with an interserotype nucleotide variability of approximately 30% [3]. Each serotype displays significant genetic diversity, categorized into well-defined groups known as *genotypes*. These genotypes are often associated with varying disease severity, ranging from mild to severe outcomes [4]. The presence of *Aedes aegypti* and *Aedes albopictus* has been documented across southern Europe, including in France (including Corsica), mainland Greece, Crete, Cyprus, Italy (including Sicily and Sardinia), the former Yugoslavia, Spain, and mainland Portugal. They are also prevalent in non-European countries around the Mediterranean basin, such as Syria, Turkey, Algeria, Egypt, Libya, Morocco, and Tunisia [5]. Notably, Europe has been increasingly affected by global warming, with rising temperatures, unusual heat waves, and extended summers creating favorable conditions for the proliferation of these competent mosquito species. Additionally, human and product movement from endemic regions has contributed to the spread of dengue in Europe. Between 2010 and 2019, autochthonous DENV-1 cases were reported in Europe, including France (2010, 2013–15, 2018, 2019), Croatia (2010), Madeira, Portugal (2012), and Spain (2018, 2019) [5,6,7,8,9].

In 2023, autochthonous DENV-1 cases were reported in Italy (82 cases), France (43 cases), and Spain (2 cases) [5]. During the summer of 2023, a DENV-1 outbreak occurred in the Lombardy region of Northern Italy in August. Cassaniti et al. [10] published preliminary findings on five patients from Lodi province (Lombardy) who tested positive for DENV-1 between 9 August and 25 August. Adult *Aedes albopictus* mosquitoes were also captured in a neighboring area on 30 August [10]. This study confirmed an autochthonous outbreak in the region. Subsequently, between August 26 and September 25, an additional eight symptomatic individuals from the same village tested positive for DENV-1. This manuscript presents a comprehensive phylogenetic and geographic analysis of the autochthonous DENV-1 outbreak in Lodi province, Northern Italy, spanning from 9 August to 25 September 2023. To date, no DENV-1 phylodynamic studies have been reported in Northern Italy. The current study addresses this gap by describing the origin and phylogeographic profile of the DENV-1 virus spread during the 2023 Lombardy outbreak.

## 2. Materials and Methods

### 2.1. Sample Collection and Sequencing

All patients presented with symptoms including fever, arthralgia, myalgia, and headache. None of these patients had traveled abroad in the past months, had no contact with people who had traveled to DENV-endemic areas, and no travel-associated cases were reported in Lodi province.

The Microbiology and Virology Department of Fondazione IRCCS Policlinico San Matteo (Pavia, Italy) analyzed 13 samples (plasma and urine) and one mosquito pool for molecular outbreak analysis (Table 1).

The first five DENV-1 consensus sequences and mosquito pool were collected from the previous work of Cassaniti et al. [10] (GenBank accession numbers: OR512925–OR512929).

Entomological inspections were carried out within a radius of 200 m around homes and places frequented by the diagnosed dengue cases, as reported in the Italian National Plan for Prevention, Surveillance, and Response to Arboviruses [5]. As a first step, sites of possible foci of larval development were mapped (manholes, storm drains, fountains, water backwaters). Monitoring activities were conducted by collecting eggs, using the ovitraps and by capturing adults with specific traps (sticky traps or BG sentinels for *Aedes albopictus*). The captured mosquitoes were immediately stored at −80 °C at the Istituto Zooprofilattico Sperimentale della Lombardia e Emilia-Romagna (IZSLER). A total of 131 *Aedes albopictus* females were sampled, pooled, and processed to detect the presence of DENV. The mosquito pools were homogenized, and viral RNA was extracted using the QIAsymphony DNA & RNA Purification System (QIAGEN, Hilden, Germany) and analyzed using the same pan-Flavivirus heminested RT-PCR targeting the NS5 gene. The RT-PCR amplification products were sequenced using Sanger sequencing. Finally, the whole-genome sequence of the pool was derived using the metagenomic approach described in Lorusso et al. [11] (Table 1).

The 8 samples collected in the period 26 August–25 September 2023 were sequenced with the proposed metagenomic approach [12] and IDseq pipeline [13] was used to perform reads quality control, alignment, and assembly to derive DENV-1 whole-genome consensus sequences (reference genome: ON123600) [14].

The collection date, specimen sources, and clinical data of patients involved in the outbreak are reported in Table 1.

The study protocol was conformed to the ethical guidelines of the Declaration of Helsinki and was approved by the Institutional Review Board and Ethical Committee of Fondazione IRCCS Policlinico San Matteo.

### 2.2. Phylogenetic Analysis

NCBI BLASTn [14,15] was exploited to perform multiple sequence alignments of Italian DENV-1 sequences with standard databases, selecting for Query Coverage and Identity Percentage from 96% to 99%. A total of 495 sequences were downloaded from the database containing the associated metadata (sampling date, host and country information).

Genotype analysis was performed on the entire dataset (495 DENV-1 sequences collected from NCBI and 14 autochthonous Italian strains) using the Genome Detective tool [16], an accurate automated system for virus classification from NGS data.

Then, 509 sequences were multiple aligned using MAFFT v7.525 [17] and visually inspected using MEGA version 11 software [18].

The maximum likelihood (ML) phylogenetic tree of the dataset was constructed using IQ-TREE multicore version 2.3.3 [19] under the GTR + G + I nucleotide substitution model selected according to BIC score (i.e., Bayesian Information Criterion), as it was the best-fitting model selected by ModelFinder [20]. The robustness of branches was evaluated using the Shimodaira–Hasegawa approximate likelihood-ratio test (SH-aLRT > 80) [21] and Ultrafast Bootstrap (UB > 80) approximation tests [22]. The phylogenetic tree was visualized using the ggtree [23] package and in-house R [24] script.

### 2.3. Similarity Analysis

The Average Nucleotide Identity (ANI) was calculated using the pwalign [25] package that solves Needleman–Wunsch pairwise (global) alignment and returns the maximum alignment score. The heatmaps were designed using the ComplexHeatmap [26] package.

### 2.4. Phylodynamic and Phylogeographic Analysis

Before coalescent analysis, the sequencing dataset was resampled to select representative and non-redundant sequences. Selection was performed using the GGRasp (Gaussian Genome Representative Selector with Prioritization) [27] package, preserving genomes of interest and minimizing the loss of genetic variation.

The temporal signals of the representative sequences (n = 252) were investigated using TempEst v.1.5.315 [28].

The BEAST v1.10.4 tool [29] was used to perform phylodynamic analysis, and the years from sample collection were used to calibrate the molecular clock. Moreover, a geographical location was assigned to each taxon based on the trait specification for each sequence. The constant coalescent Bayesian method was exploited with a relaxed uncorrelated molecular clock using time-stamped data scaled in years and a GTR + G + I nucleotide substitution model (recovered from ModelFinder analysis [20]).

Parameter estimates were obtained from the MCMC run of 15 × 10^7^ generation and a sampling frequency of 103. The performance of the transition kernel was inspected, and the acceptance ratio was greater than 0.234. The convergence of parameters was inspected with Tracer [30] v.1.7.2 which estimated the Effective Sample Size (ESS) (i.e., measurement of the number of effectively independent samples in each run). The analysis was considered to have converged and reached stability after the burn-in period (10%) when ESS was higher than 150. The trees sampled at every 10,000 steps were summarized in a maximum clade credibility (MCC) tree with 10% burn-in using TreeAnnotator [29] and visualized using FigTree v.1.4.4 [31].

The MCC tree was visualized using the ggtree [23] package and in-house R [24] script. To determine the epidemiological characteristics of the DENV-1 dataset, the geographic transmission pattern was reconstructed using BEAST software [29]. The delineation of significant migration paths among Italian strains was validated using country rate posterior probability (PP) values (PP ≥ 0.5). The map and migration paths were built and visualized using SPREAD3 software version 0.9.6 [32] while the countries’ geographic coordinates were recovered using the Geoapify website [33].

## 3. Results

The current study analyzed 14 cases from the 2023 Italian Dengue outbreak. The samples sequenced in this study (n = 8) were collected between 26 August and 25 September 2023 and sequenced with NGS approach as reported in Section 2.

However, considering that the outbreak officially started on 9 August, we retrieved the sequences of the first 5 DENV-1 cases collected between 9 and 25 August and the mosquito pool from Cassaniti et al. [10] (accession numbers: OR512925–OR512929) (Table 1).

In the first period of the outbreak, the patients were three males and two females, with an average age of 53.2 years (ranging from 3 to 88); while the second group, collected between 26 August and 25 September, consisted of eight symptomatic individuals, four males, and four females, with an average age of 46 years (spanning from 16 to 68). All patients exhibited symptoms such as fever, joint pain, muscle pain, and headaches, with none having traveled in the past month, as shown in Table 1.

The mosquito pool sequence derived from 131 females *Aedes albopictus* captured within a radius of 200 m around homes and places frequented by the diagnosed dengue cases (Table 1) [10,34].

### 3.1. Similarity Analysis

**Figure 1:**
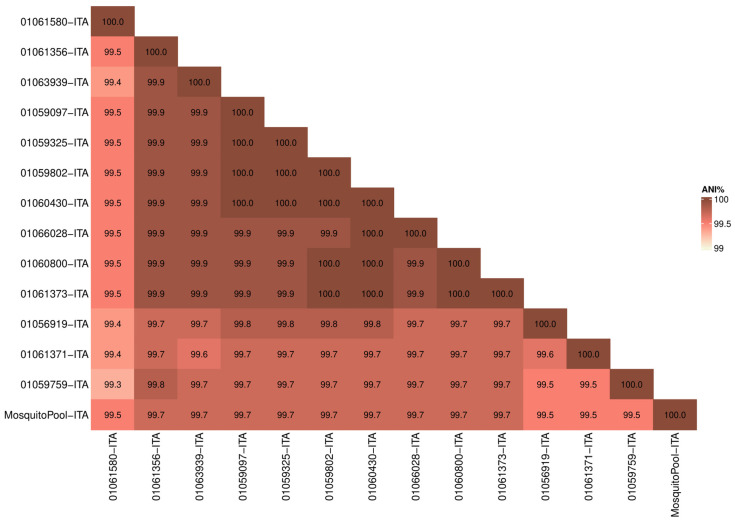
Average Nucleotide Identity (ANI). Heatmap showing nucleotide similarity of DENV-1 complete CDS sequences between Italian outbreak strains. Red and white shadings represent higher (100%) and lower (99%) relative ANI percentages, respectively.

Whole-genome sequences were first analyzed to determine similarity in terms of nucleotide identity among samples. The Average Nucleotide Identity (ANI) among the 14 strains (13 patients and one mosquito pool) resulted in 99.7% (range 99.3–100) confirming the high similarity of the outbreak strains (Figure 1). Moreover, the mosquito pool had ANI values ranging from 99.5 to 99.7 compared to clinical samples (Figure 1).

The analysis confirmed that the virus found in mosquitoes captured in Lodi province was transmitted to patients positive for DENV-1 (Table 1).

### 3.2. Phylogenetic Analysis

Multiple sequence alignments of Italian DENV-1 sequences with NCBI standard databases were performed, selecting sequences for Query Coverage and Identity Percentage from 96% to 99% [14,15]. The final dataset included 495 whole-genome DENV-1 sequences with associated metadata (sampling date, host, and country information) and 14 Italian sequences presented in this study.

**Figure 2:**
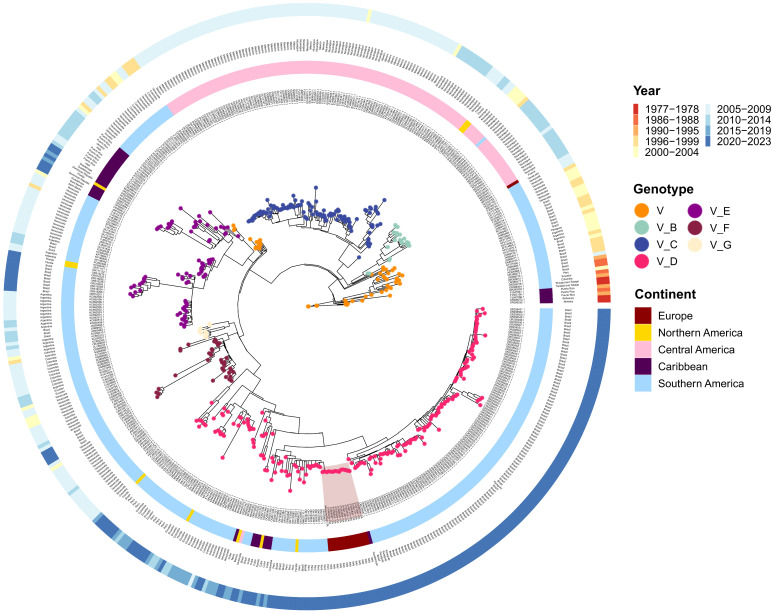
Phylogenetic ML tree of DENV-1 complete genome sequences. The inner layer contains strain names, the second layer represents the origin continent followed by country names (third layer). The outer layer contains sampling dates divided in 4-year blocks. Tree nodes classify strain genotype and subtypes. Italian strains (n = 14) from the August–September 2023 outbreak are highlighted in dark red. ML: maximum likelihood.

Subsequent analyses were performed by extracting the DENV-1 coding DNA sequence (CDS) and removing the start and final UTRs. The CDS of the DENV-1 dataset (n = 509) ranged in size from 9064 to 10,185 nucleotides with various topographical backgrounds, including North, Central, and South America, the Caribbean, and Europe (Figure 2, Table 2 and Table S1). However, the mean length of the open reading frame (~10,157.1 nucleotide) was similar across the DENV-1 strains (Supplementary Table S1).

From a geographical standpoint, the dataset included 509 sequences, including 15 from Europe (~3%), 10 from North America (~2%), 131 from Central America (~26%), 31 from the Caribbean (~6%), and 322 from South America (~63%) (Table 2, Figure 2).

Sequencing time ranged from 1977 to 2023, with the most abundant samples in 2020–2023 (n = 183, ~36%) and 2005–2009 (n = 177, ~35%). The others were distributed as follows: 2010–2014 (n = 60, ~12%), 2000–2004 (n = 30, ~6%), 2015–2019 (n = 24, ~5%), 1996–1999 (n = 23, ~4.5%), 1990–1995 (n = 4, ~1%), 1986–1988 (n = 4, ~1%), 1977–1978 (n = 4, ~1%) (Table 2, Figure 2).

Maximum likelihood analysis of DENV-1 revealed a clear separation of genotype V and its division into six subtypes (Figure 2). However, for 51 strains, genotype was defined but not subtype. Genotype V subtype D was most prevalent with 191 strains out of 509 (including Italian strains, red highlight, Figure 2). Moreover, subtype D strains were predominantly found in South America and Europe, followed by Caribbean and North America. South America also contained subtype E strains (n = 58), shared with the Caribbean (n = 18), and exclusively subtypes F (n = 39) and G (n = 11). Strains from Central America belonged to subtypes B (n = 17) and C (n = 113) (Figure 2, Supplementary Table S1).

**Figure 3:**
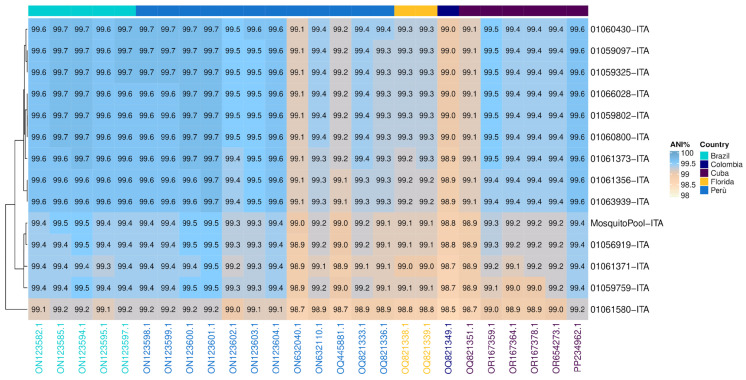
Average Nucleotide Identity (ANI). Heatmap showing nucleotide similarity of DENV-1 complete CDS sequences. The comparison was made between Italian outbreak strains vs. 26 phylogenetically nearby strains (see Figure 2): Brazil (n = 6), Peru (n = 12), Florida (n = 2), Colombia (n = 1), Cuba (n = 6). Strains on columns were arranged in clusters based on countries of origin. Sky blue and white shadings represent higher (100%) and lower (98%) relative ANI percentages, respectively. CDS: coding DNA sequence.

Similarity analysis was performed between Italian strains (red highlight, Figure 2) and the 26 nodes of the tree that belonged to the same monophyletic group (clade) (Figure 3). The clade comprised 14 autochthonous Italian strains, 5 Brazilian, 12 Peruvian, two from Florida, one Colombian, and 6 from Cuba. Overall, the ANI calculated for the clade resulted in 99.3% (range: 98.5–99.7) (Figure 3). However, the 14 autochthonous Italian strains clustered with South American strains (Peruvian and Brazilian) collected from 2020 to 2023, with an Average Nucleotide Identity ranging from 99.6 to 99.7 among DENV-1 strains of genotype V subtype D (Figure 3).

### 3.3. Phylodynamic and Phylogeographic Diffusion

To determine which lineages circulated in Italy and were associated with the outbreak in the space-time dimension, the phylodynamic and phylogeographic diffusion of the dataset were studied. However, the number of genomes available in the dataset for comparative genomic analysis and the relative genome redundancy (i.e., high similarity) can make the phylodynamic analysis process extremely slow. Therefore, a reduction in genome redundancy is necessary to maximize diversity. Thus, the entire dataset was resampled to select strains using the GGrasp package [27]. The aim was to generate and return a representative set of genomes from a large dataset with a defined origin. As a result, we obtained a non-redundant representative dataset that preserved the genomes of interest while minimizing the loss of genetic variation [27].

The final dataset of representative sequences (n = 224) contained strains as described in Table 3. Overall, the selection affected more Central (10.7%) and South America (66.9%) strains and fewer Caribbean (11.6%) and North America (4%), leaving unchanged the number of strains in Europe (15).

The temporal signal of the representative sequences was calculated to confirm the presence of sufficient genetic change between sampling times (R^2^ = 0.92, correlation coefficient = 0.96) [28].

The time to the most recent common ancestor (tMRCA) of the DENV-1 dataset was estimated using the Bayesian Markov chain Monte Carlo method implemented in BEAST v1.10.4 [29].

**Figure 4:**
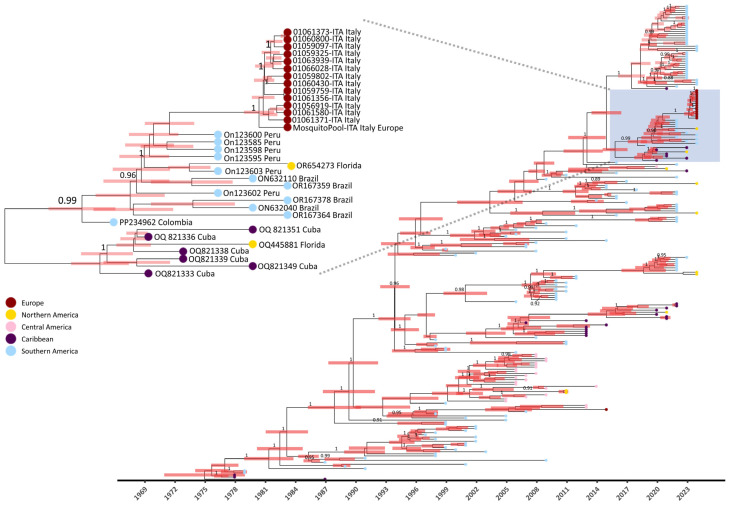
MCC tree for dengue virus serotype 1 in study of transmission dynamics of dengue. The MCC tree was constructed using the set of representative DENV-1 sequences (n = 252) elaborated with BEAST software [29]. Posterior probabilities ≥ 0.85 are shown in internal nodes and 98% HPD intervals are shown as red bars. The box highlights Italian strains clustering with Peruvian and Brazilian strains. CDS: coding DNA sequence; HPD: highest posterior density; MCC: maximum clade credibility.

The Bayesian analysis estimated a mean evolutionary substitution rate of 8.234 × 10^−4^ subs/site/years (95% HPD (highest posterior density) interval: 7.1448 × 10^−4^–9.3343 × 10^−4^). The time to the most recent common ancestor (tMRCA) estimate was 1972 (95% HPD interval: 1968–1976) with a mean age of 50 years (95% HPD interval: 46.9–54.9). The time of divergence of Italian strains from Peruvian and Brazilian strains was estimated to have occurred approximately in September 2020 (range: January 2020–June 2021) (Figure 4).

**Figure 5:**
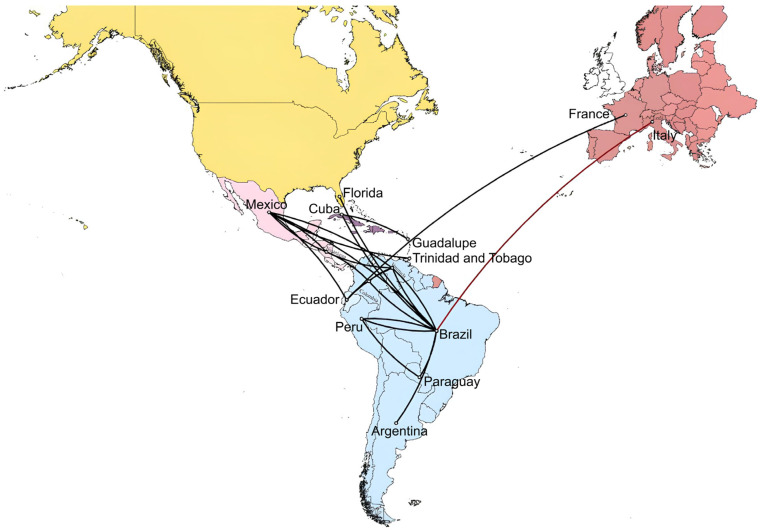
Phylogeographic diffusion of DENV-1 across Americas, Caribbean, and Europe through spatial projection of the MCC tree generated in BEAST software [29]. The lines represent branches connecting geographic locations. The dark red line color indicates the last projection from Brazil to Lodi province where the Italian outbreak occurred. Significant non-zero rates were selected considering a country rate PP threshold (PP ≥ 0.5). MCC: maximum clade credibility, PP: posterior probability.

To reconstruct the spatial diffusion of DENV-1 serotypes across countries, estimate the ancestral locations of the virus, and simultaneously infer the history of country jumping, discrete phylogeographic analyses using maximum clade credibility (MCC) trees were performed for the DENV-1 dataset (Figure 5). The delineation of significant migration paths was validated on thresholds of country rate PP values, as described in Section 2. The analysis revealed that the migration of strains in this dataset spread around 1980 from the Caribbean (Trinidad and Tobago), moved to Central America (~1985), and finally to South America from 1985 to 1990s. Until 2005, strains have circulated in several South American countries (Brazil, Peru, Venezuela, Colombia, Ecuador, and Argentina). Between 2005 and 2015, strains migrated between South and Central America. The first presumable migration from Ecuador to Europe, particularly France, occurred between 2014 and 2015. The most likely migration of strains from Brazil to Italy was in the last months of 2022, which triggered the 2023 outbreak (Figure 5).

## 4. Discussion

The incidence of DENV has increased dramatically worldwide in recent decades.

DENV is prevalent in 128 countries across South and Southeast Asia, the Caribbean, Central and South America, and Africa, infecting 50 to 100 million people annually, with approximately half a million needing hospital treatment and resulting in 12,500 to 25,000 fatalities [5]. Regarding Europe, the GenBank public repository reveals that three dengue serotypes (DENV-1, DENV-2, and DENV-3) have circulated in Italy between 2015 and 2023 with DENV1 as the predominant serotype reported [5,14]. In Italy, a recent trend is emerging in line with global developments seen since early 2023, which include an upsurge in the transmission of DENV-1 and DENV-3 viruses [35,36].

Despite the increasing number of DENV-1 cases in Europe in recent years, few studies have examined the origin and dynamics. In Italy, surveillance activities as part of public health practice (the Italian National Plan for Prevention, Surveillance, and Response to Arboviruses [34]) carried out by health institutions allow physicians and researchers to monitor vector-borne viruses and related diseases for potential outbreaks. This study analyzed the phylogeny and phylogeography of DENV-1 in Northern Italy, highlighting the dynamics of introduction, circulation, and expansion of a strain of the virus. The results showed a common local origin in the province of Lodi with 99.7% similarity between all sequences retrieved from patients and *Aedes albopictus* mosquitoes. The high similarity between the strains retrieved from DENV patients and the one retrieved from the mosquito pool confirmed the common local origin of the outbreak in Lodi province.

The progressive evolution of DENV strains has been observed over time, with possible consequences, such as serotype immunological cross-reaction and vector competence modification (e.g., *Aedes albopictus* or *Aedes aegypti*).

Moreover, climate changes significantly altered DENV vector competence in Europe. The literature revealed that both *Aedes aegypti* and *Aedes albopictus* infested Europe throughout the entire period (2006–2015) [37]. One reason that DENV-1 transmission has unleashed the outbreak is the circulation of competent *Aedes albopictus* in Pianura Padana areas containing large amounts of water due to rice production (i.e., mosquito larvae can only live and develop in water). However, one of the main determinants of DENV transmission is human mobility because the flight range of *Aedes* mosquitoes is minimal [38]. The movement of symptomatic or asymptomatic subjects may promote the spread of the virus to other regions, increasing the risk of rapid DENV spread. Indeed, the displacement of humans worldwide has increased in recent years after SARS-CoV-2 infection, leading to a higher probability of importing cases from endemic countries.

Since 2010, when the first dengue outbreak was recorded in Europe, 48 DENV outbreaks have occurred in many countries (Austria, Bulgaria, Croatia, France, Germany, Greece, Hungary, Italy, Malta, Portugal, Romania, Slovenia) [5].

DENV outbreaks are usually identified and monitored through serological tests, reverse transcription-polymerase chain reaction (RT-PCR), using generic flavivirus primers targeting the non-structural protein 5 (NS5) gene. However, new sequencing approaches (NGS) that allow the determination of whole-genome sequences are proving decisive in defining the spread of arboviruses both in Europe and globally.

Indeed, between 2010 and 2019, instances of autochthonous DENV-1 cases in Europe were documented both serologically and by sequencing in France [6], Croatia [7], and Portugal [5,8]. In this study, the use of NGS allowed us to infer the derivation of the DENV-1 Italian outbreak from strains that spread in South America (Brazil and Peru) between 2015 and 2022 (Figure 4 and Figure 5). Moreover, our findings are consistent with South American local epidemiology since Italian strains belonged to a monophyletic cluster of genotype V (Figure 2 and Figure 3). Our data also suggest that DENV-1 was introduced in Northern Italy after 2019 (Figure 4, tMRCA of Italian strains: 2019).

Determining the adaptation dynamics of these strains in Lombardy remains challenging, as isolated cases recorded between 2019 and 2023 may have been misclassified as imported from abroad rather than stemming from autochthonous transmission. Furthermore, the existing surveillance system has not provided sufficient data to gain a more detailed understanding of the evolutionary patterns and origins of DENV-1 cases in Italy.

The detection of an autochthonous case in Europe should trigger epidemiological and entomological investigations to assess the size of the transmission area and the potential for onward transmission and to guide vector control measures.

For all these reasons, Italy has activated a national surveillance and control plan for arboviruses, extending from 2020 to 2025, to counteract an important public health threat [34]. Several other issues need to be studied to better define the epidemiological scenario in which DENV is circulating worldwide and how the evolution of this virus may modify the competence of the vector and the pathogenesis of infection.

## Figures and Tables

**Table 1:** Italian DENV-1 cases occurred in Lodi province in the period 9 August to 25 September 2023. For each sample, accession number, collection date, specimen source, gender, and age was reported. The first five samples and the mosquito pool were collected from the paper of Cassaniti et al. [10].

Strain ID	GenBank/GISAID Accession Numbers	Collection Date	Specimen Source	Gender	Age
01056919-ITA	OR512929 [10]	9 August 2023	Urine	Male	72
01059097-ITA	OR512927 [10]	22 August 2023	Plasma	Male	88
01059325-ITA	OR512928 [10]	23 August 2023	Plasma	Female	3
01059759-ITA	OR512925 [10]	25 August 2023	Plasma	Female	82
01059802-ITA	OR512926 [10]	25 August 2023	Plasma	Male	21
Mosquitoes pool [10]	-	30 August 2023	*Aedes albopictus*	Female	-
01060430-ITA	EPI ISL 19216783	29 August 2023	Plasma	Male	16
01060800-ITA	EPI ISL 19216784	31 August 2023	Plasma	Female	41
01061356-ITA	EPI ISL 19216785	4 September 2023	Plasma	Male	42
01061371-ITA	EPI ISL 19216786	4 September 2023	Plasma	Female	53
01061373-ITA	EPI ISL 19216787	4 September 2023	Plasma	Female	62
01061580-ITA	EPI ISL 19216788	5 September 2023	Plasma	Female	38
01063939-ITA	EPI ISL 19216789	15 September 2023	Plasma	Male	68
01066028-ITA	EPI ISL 19216790	25 September 2023	Plasma	Male	48

**Table 2:** DENV-1 complete dataset. The table reports the number of total strains for each continent in the first column. The number of relative samples for each country and sampling date are reported in brackets.

Number of Strains	Continent	Country	Sampling Date
15	Europe	Italy (14)	2020–2023 (14)
		France (1)	2010–2014 (1)
10	North America	Florida (9)	2010–2014 (2) 2020–2023 (7)
		Texas (1)	2020–2023 (1)
			2000–2004 (6)
		Nicaragua (62)	2005–2009 (30)
131	Central America		2010–2014 (26)
		Mexico (69)	2005–2009 (68)
			2015–2019 (1)
			1986–1988 (1)
		Puerto Rico (11)	1990–1995 (2) 2005–2009 (2)
			2010–2014 (6)
		Haiti (2)	2010–2014 (2)
		Jamaica (2)	1977–1978 (1)
31	Caribbean		2010–2014 (1)
		Saint Martin (2)	2020–2023 (2)
		Cuba (7)	2015–2019 (2)
			2020–2023 (5)
		Dominican Republic (2)	2015–2019 (2)
		Guadeloupe (4)	2020–2023 (4)
		Bahamas (1)	1977–1978 (1)
			1986–1988 (2)
			1990–1995 (1)
			1996–1999 (9)
		Brazil (148)	2000–2004 (6)
			2005–2009 (2)
			2010–2014 (6)
			2020–2023 (122)
			2000–2004 (3)
		Argentina (24)	2005–2009 (16)
			2010–2014 (5)
		Paraguay (5)	2000–2004 (1)
			2020–2023 (4)
			1996–1999 (9)
			2000–2004 (11)
		Venezuela (75)	2005–2009 (47)
322	South America		2010–2014 (4)
			2015–2019 (4)
			1990–1995 (1)
			1996–1999 (5)
			2000–2004 (2)
		Colombia (27)	2005–2009 (11)
			2010–2014 (1)
			2015–2019 (5)
			2020–2023 (2)
			1986–1988 (1)
		Ecuador (18)	2005–2009 (1) 2010–2014 (6)
			2015–2019 (10)
		Peru (23)	2000–2004 (1)
			2020–2023 (22)
		Trinidad and Tobago (2)	1977–1978 (2)

**Table 3:** Composition of DENV-1 dataset after selection of representative sequences. The table reports the continent (total number in brackets), the sampling date, and the number of strains, respectively.

Continent	Sampling Date	Number of Strains
Europe (15)	2020–2023	14
	2010–2014	1
North America (9)	2010–2014	2
	2020–2023	7
Central America (24)	2000–2004	2
	2005–2009	18
	2010–2014	3
	2015–2019	1
Caribbean (26)	1977–1978	2
	1986–1988	1
	2005–2009	1
	2010–2014	7
	2015–2019	4
	2020–2023	11
South America (150)	1977–1978	2
	1986–1988	2
	1990–1995	2
	1996–1999	17
	2000–2004	14
	2005–2009	21
	2010–2014	15
	2015–2019	9
	2020–2023	68

## Data Availability

Sequence data generated in this study (8 DENV-1 WGS samples) have been submitted to GISAID [39] under accession numbers: EPI _ISL_19216790, EPI _ISL_19216784, EPI _ISL_19216789, EPI_ISL_19216786, EPI _ISL_19216785, EPI _ISL_19216783, EPI _ISL_19216788, EPI_ISL_19216787.

## Supplementary Materials

The following supporting information can be downloaded at https://www.mdpi.com/article/10.3390/pathogens13121124/s1, Table S1. DENV-1 strains.

## Abbreviations

ANI	Average Nucleotide Identity
BIC	Bayesian Information Criterion
CDS	Coding DNA Sequence
DENV-1	Dengue Virus type 1
ESS	Effective Sample Size
HPD	Highest Posterior Density
IZSLER	Istituto Zooprofilattico Sperimentale della Lombardia e Emilia-Romagna
ML	Maximum Likelihood
MCC	Maximum Clade Credibility MCM = Markov Chain Monte Carlo
ORF	Open Reading Frame
SH-aLRT	Shimodaira–Hasegawa approximate Likelihood-Ratio Test
tMRCA	time to the Most Recent Common Ancestor
UB	Ultrafast Bootstrap approximation test
