# Supplementary material for: Tracing the Origin, Spread, and Molecular Evolution of Dengue Type 1 Cases That Occurred in Northern Italy in 2023
Source: Pathogens. 2024 Dec 19;13(12):1124. doi: 10.3390/pathogens13121124 (PMC11678104; doi:10.3390/pathogens13121124)
Supplement: Supplementary file 1 [file pathogens-13-01124-s001.zip › pathogens-3315681-supplementary.pdf]

Strain ID	Continent	Country	Sampling_date	CDS length	Genotype_Subtype
01056919-ITA	Europe	Italy	2023	10118	V_D
01059097-ITA	Europe	Italy	2023	10179	V_D
01059325-ITA	Europe	Italy	2023	10179	V_D
01059759-ITA	Europe	Italy	2023	10119	V_D
01059802-ITA	Europe	Italy	2023	10179	V_D
01060430-ITA	Europe	Italy	2023	10179	V_D
01060800-ITA	Europe	Italy	2023	10179	V_D
01061356-ITA	Europe	Italy	2023	10179	V_D
01061371-ITA	Europe	Italy	2023	10185	V_D
01061373-ITA	Europe	Italy	2023	10179	V_D
01061580-ITA	Europe	Italy	2023	9064	V_D
01063939-ITA	Europe	Italy	2023	10179	V_D
01066028-ITA	Europe	Italy	2023	10179	V_D
AB519681.1	Southern America	Brazil	2001	10179	V
AF226685.2	Southern America	Brazil	1990	10179	V
AF311956.1	Southern America	Brazil	1997	10179	V
AF311957.1	Southern America	Brazil	1997	10179	V
AF311958.1	Southern America	Brazil	1997	10179	V
AF513110.1	Southern America	Brazil	2001	10179	V
AF514876.3	Southern America	Argentina	2000	10179	V
AF514883.2	Southern America	Paraguay	2000	10179	V
AF514885.3	Southern America	Argentina	2000	10179	V
AY277665.2	Southern America	Argentina	2000	10179	V
EU482591.1	Caribbean	Puerto Rico	2006	10179	V_E
EU482609.1	Southern America	Venezuela	2007	10179	V_F
EU482610.1	Southern America	Venezuela	2007	10179	V_E
EU482611.1	Southern America	Venezuela	2007	10179	V_E
EU482615.1	Central America	Nicaragua	2005	10179	V_C
EU482616.1	Central America	Nicaragua	2005	10179	V_C
EU482617.1	Central America	Nicaragua	2005	10179	V_C
EU482618.1	Central America	Nicaragua	2005	10179	V_C
EU482619.1	Central America	Nicaragua	2005	10179	V_C
EU596501.1	Central America	Nicaragua	2004	10179	V_C
EU596503.1	Central America	Nicaragua	2005	10179	V_C
EU596504.1	Central America	Nicaragua	2005	10179	V_C
FJ024423.1	Central America	Nicaragua	2005	10179	V_C
FJ024479.1	Central America	Nicaragua	2006	10179	V_C
FJ024480.1	Central America	Nicaragua	2005	10179	V_C
FJ024481.1	Central America	Nicaragua	2005	10179	V_C
FJ024482.1	Central America	Nicaragua	2005	10179	V_C
FJ024483.1	Central America	Nicaragua	2005	10179	V_C
FJ024484.1	Central America	Nicaragua	2005	10179	V_C
FJ024485.1	Central America	Nicaragua	2005	10179	V_C
FJ182002.1	Central America	Nicaragua	2005	10179	V_C
FJ384655.1	Southern America	Brazil	2008	10179	V
FJ410290.1	Central America	Nicaragua	2005	10179	V_C
FJ547068.1	Central America	Nicaragua	2006	10179	V_C
FJ547086.1	Caribbean	Puerto Rico	1995	10179	V
FJ547087.1	Caribbean	Puerto Rico	1992	10179	V
FJ547088.1	Central America	Nicaragua	2008	10179	V_C
FJ547089.1	Central America	Nicaragua	2005	10179	V_C
FJ562104.1	Central America	Nicaragua	2006	10179	V_C

FJ562106.1	Caribbean	Puerto Rico	1986	10179	V
FJ639735.1	Southern America	Venezuela	1997	10179	V_B
FJ639740.1	Southern America	Venezuela	1998	10179	V
FJ639741.1	Southern America	Venezuela	1998	10179	V
FJ639743.1	Southern America	Venezuela	1999	10179	V
FJ639794.1	Southern America	Venezuela	2004	10179	V_F
FJ639796.1	Southern America	Venezuela	2004	10179	V_F
FJ639797.1	Southern America	Venezuela	2004	10179	V_F
FJ639802.1	Southern America	Venezuela	2004	10179	V_F
FJ639806.1	Southern America	Venezuela	2007	10179	V_F
FJ639808.1	Southern America	Venezuela	2005	10179	V_F
FJ639811.1	Southern America	Venezuela	2005	10179	V
FJ639812.1	Southern America	Venezuela	2005	10179	V_F
FJ639813.1	Southern America	Venezuela	2005	10179	V_F
FJ639814.1	Southern America	Venezuela	2005	10179	V_E
FJ639815.1	Southern America	Venezuela	2006	10179	V_E
FJ639818.1	Southern America	Venezuela	2006	10179	V_F
FJ639819.1	Southern America	Venezuela	2006	10179	V_E
FJ639820.1	Southern America	Venezuela	2006	10179	V_F
FJ639821.1	Southern America	Venezuela	2006	10179	V
FJ639823.1	Southern America	Venezuela	2006	10179	V_F
FJ639824.1	Southern America	Venezuela	2006	10179	V_F
FJ744701.1	Southern America	Venezuela	2004	10179	V_F
FJ810415.1	Southern America	Venezuela	2005	10179	V_E
FJ810419.1	Central America	Nicaragua	2006	10179	V_C
FJ850093.1	Southern America	Brazil	2008	10179	V_F
FJ850099.1	Southern America	Venezuela	2007	10179	V_F
FJ850100.1	Southern America	Venezuela	2007	10179	V_F
FJ850101.1	Southern America	Venezuela	2007	10179	V_F
FJ850102.1	Southern America	Venezuela	2007	10179	V_E
FJ850103.1	Southern America	Venezuela	2008	10179	V_E
FJ850104.1	Southern America	Venezuela	2008	10179	V
FJ850113.1	Central America	Nicaragua	2005	10179	V_C
FJ850114.1	Central America	Nicaragua	2005	10179	V_C
FJ873809.1	Southern America	Venezuela	2007	10179	V_F
FJ873810.1	Southern America	Venezuela	2007	10179	V_E
FJ873814.1	Central America	Nicaragua	2005	10179	V_C
FJ882579.1	Southern America	Venezuela	2007	10179	V_F
FJ898433.1	Central America	Nicaragua	2007	10179	V_C
FJ898437.1	Central America	Nicaragua	2004	10179	V_C
GQ199857.1	Central America	Nicaragua	2008	10179	V_C
GQ199858.1	Central America	Nicaragua	2008	10179	V_C
GQ199859.1	Central America	Nicaragua	2008	10179	V_B
GQ199867.1	Central America	Nicaragua	2004	10179	V_C
GQ199872.1	Central America	Nicaragua	2004	10179	V_C
GQ199873.1	Central America	Nicaragua	2004	10179	V_C
GQ199875.1	Central America	Nicaragua	2004	10179	V_C
GQ199877.1	Southern America	Venezuela	2007	10179	V_E
GQ868498.1	Central America	Mexico	2006	10179	V_C
GQ868499.1	Central America	Mexico	2006	10179	V_C
GQ868500.1	Central America	Mexico	2007	10179	V_C
GQ868501.1	Central America	Mexico	2007	10179	V_C
GQ868502.1	Central America	Mexico	2007	10179	V_C

GQ868503.1	Central America	Mexico	2007	10179 V_C
GQ868504.1	Central America	Mexico	2007	10179 V_C
GQ868505.1	Central America	Mexico	2007	10179 V_C
GQ868506.1	Central America	Mexico	2007	10179 V_C
GQ868507.1	Central America	Mexico	2007	10179 V_C
GQ868508.1	Central America	Mexico	2007	10179 V_C
GQ868509.1	Central America	Mexico	2007	10179 V_C
GQ868510.1	Central America	Mexico	2007	10179 V_C
GQ868511.1	Central America	Mexico	2007	10179 V_C
GQ868512.1	Central America	Mexico	2007	10179 V_C
GQ868513.1	Central America	Mexico	2007	10179 V_C
GQ868514.1	Central America	Mexico	2007	10179 V_C
GQ868517.1	Central America	Mexico	2007	10179 V_C
GQ868518.1	Central America	Mexico	2007	10179 V_C
GQ868519.1	Central America	Mexico	2007	10179 V_C
GQ868520.1	Central America	Mexico	2007	10179 V_C
GQ868521.1	Central America	Mexico	2007	10179 V_C
GQ868522.1	Central America	Mexico	2007	10179 V_C
GQ868523.1	Central America	Mexico	2007	10179 V_C
GQ868524.1	Central America	Mexico	2007	10179 V_C
GQ868525.1	Central America	Mexico	2007	10179 V_C
GQ868526.1	Central America	Mexico	2007	10179 V_C
GQ868527.1	Central America	Mexico	2007	10179 V_C
GQ868528.1	Central America	Mexico	2007	10179 V_C
GQ868529.1	Central America	Mexico	2008	10179 V_C
GQ868530.1	Central America	Mexico	2008	10179 V_C
GQ868531.1	Central America	Mexico	2008	10179 V_C
GQ868532.1	Central America	Mexico	2008	10179 V_C
GQ868533.1	Central America	Mexico	2008	10179 V_C
GQ868534.1	Central America	Mexico	2008	10179 V_C
GQ868535.1	Central America	Mexico	2008	10179 V_C
GQ868536.1	Central America	Mexico	2008	10179 V_C
GQ868537.1	Central America	Mexico	2008	10179 V_C
GQ868538.1	Central America	Mexico	2008	10179 V_C
GQ868539.1	Central America	Mexico	2008	10179 V_C
GQ868559.1	Southern America	Colombia	1998	10179 V
GQ868560.1	Southern America	Colombia	1998	10179 V
GQ868561.1	Southern America	Colombia	1999	10179 V
GQ868562.1	Southern America	Colombia	2005	10179 V_F
GQ868563.1	Southern America	Colombia	2006	10179 V_G
GQ868564.1	Southern America	Colombia	2006	10179 V_G
GQ868565.1	Southern America	Colombia	2006	10179 V_G
GQ868566.1	Southern America	Colombia	2007	10179 V_G
GQ868567.1	Southern America	Colombia	2007	10179 V_G
GQ868568.1	Southern America	Colombia	2007	10179 V_G
GQ868569.1	Southern America	Colombia	2007	10179 V_G
GQ868570.1	Southern America	Colombia	2008	10179 V_D
GU056029.1	Southern America	Venezuela	1997	10179 V_E
GU056030.1	Southern America	Venezuela	1997	10179 V
GU056031.1	Southern America	Venezuela	1998	10179 V_E
GU056032.1	Southern America	Venezuela	1998	10179 V_C
GU056033.1	Southern America	Venezuela	1998	10179 V
GU131832.1	Southern America	Venezuela	2000	10179 V_E

GU131833.1	Southern America	Venezuela	2000	10179	V
GU131834.1	Southern America	Venezuela	2001	10179	V
GU131835.1	Southern America	Venezuela	2004	10179	V
GU131836.1	Southern America	Venezuela	2004	10179	V
GU131837.1	Southern America	Venezuela	2005	10179	V
GU131838.1	Southern America	Venezuela	2006	10179	V_E
GU131839.1	Southern America	Venezuela	2006	10179	V_E
GU131840.1	Southern America	Venezuela	2007	10179	V_E
GU131841.1	Southern America	Venezuela	2007	10179	V_E
GU131842.1	Southern America	Venezuela	2007	10179	V_F
GU131948.1	Southern America	Colombia	2001	10179	V
GU131949.1	Southern America	Colombia	2006	10179	V_G
GU131956.1	Central America	Mexico	2006	10179	V_C
GU131957.1	Central America	Mexico	2006	10179	V_C
GU131958.1	Central America	Mexico	2006	10179	V_C
GU131960.1	Central America	Mexico	2007	10179	V_C
GU131961.1	Central America	Mexico	2007	10179	V_C
GU131962.1	Central America	Mexico	2007	10170	V_C
GU131964.1	Central America	Mexico	2007	10179	V_C
GU131965.1	Central America	Mexico	2007	10179	V_C
GU131966.1	Central America	Mexico	2007	10179	V_C
GU131969.1	Central America	Mexico	2007	10179	V_C
GU131970.1	Central America	Mexico	2007	10179	V_C
GU131971.1	Central America	Mexico	2007	10179	V_C
GU131972.1	Central America	Mexico	2007	10179	V_C
GU131977.1	Central America	Mexico	2007	10179	V_C
GU131978.1	Central America	Mexico	2007	10179	V_C
GU131979.1	Central America	Mexico	2007	10179	V_C
GU131980.1	Central America	Mexico	2007	10179	V_C
GU131981.1	Central America	Mexico	2007	10179	V_C
GU131982.1	Central America	Mexico	2008	10179	V_C
GU131983.1	Central America	Mexico	2008	10179	V_C
GU131984.1	Central America	Mexico	2008	10179	V_C
HM631855.1	Central America	Mexico	2007	10177	V_C
HQ166035.1	Central America	Mexico	2007	10179	V_C
HQ166036.1	Central America	Mexico	2007	10179	V_C
HQ166037.1	Central America	Mexico	2008	10179	V_C
HQ332177.1	Southern America	Venezuela	2006	10179	V_F
HQ332179.1	Southern America	Venezuela	2007	10179	V_F
HQ332180.1	Southern America	Venezuela	2006	10179	V_F
HQ332181.1	Southern America	Venezuela	2006	10179	V_E
HQ332182.1	Southern America	Venezuela	2006	10179	V_B
HQ332183.1	Southern America	Venezuela	2007	10179	V_B
JN819402.1	Central America	Nicaragua	2005	10179	V_C
JN819405.1	Southern America	Venezuela	2006	10179	V_F
JN819410.1	Southern America	Venezuela	2005	10179	V_E
JN819411.1	Southern America	Venezuela	2005	10179	V_F
JN819412.1	Southern America	Venezuela	2005	10179	V_F
JN819413.1	Southern America	Venezuela	2006	10179	V_F
JN819414.1	Southern America	Venezuela	2007	10179	V_F
JN819415.1	Southern America	Venezuela	2006	10179	V_B
JN819425.1	Southern America	Venezuela	2004	10179	V_E
JQ675358.1	Northern America	Florida	2010	10179	V_C

JX669462.1	Southern America	Brazil	2010	10179	V_E
JX669463.1	Southern America	Brazil	2010	10179	V_E
JX669466.1	Southern America	Brazil	2010	10179	V_E
JX669467.1	Southern America	Brazil	1996	10179	V
JX669468.1	Southern America	Brazil	1997	10179	V
JX669469.1	Southern America	Brazil	1997	10179	V
JX669470.1	Southern America	Brazil	1998	10179	V
JX669471.1	Southern America	Brazil	1999	10179	V
JX669472.1	Southern America	Brazil	2000	10179	V
JX669473.1	Southern America	Brazil	2001	10179	V
JX669474.1	Southern America	Brazil	2001	10179	V
JX669475.1	Southern America	Brazil	2002	10179	V
KC692495.1	Southern America	Argentina	2009	10179	V_E
KC692496.1	Southern America	Argentina	2009	10179	V_E
KC692497.1	Southern America	Argentina	2009	10179	V_E
KC692498.1	Southern America	Argentina	2009	10179	V_E
KC692499.1	Southern America	Argentina	2009	10179	V_E
KC692500.1	Southern America	Argentina	2009	10179	V_E
KC692501.1	Southern America	Argentina	2009	10179	V_E
KC692502.1	Southern America	Argentina	2009	10179	V_E
KC692503.1	Southern America	Argentina	2009	10179	V_E
KC692504.1	Southern America	Argentina	2009	10179	V_E
KC692505.1	Southern America	Argentina	2009	10179	V_E
KC692506.1	Southern America	Argentina	2009	10179	V_E
KC692507.1	Southern America	Argentina	2009	10179	V_E
KC692508.1	Southern America	Argentina	2009	10179	V_E
KC692509.1	Southern America	Argentina	2009	10179	V_E
KC692510.1	Southern America	Argentina	2009	10179	V_E
KC692512.1	Southern America	Argentina	2010	10179	V_E
KC692514.1	Southern America	Argentina	2010	10179	V_E
KC692515.1	Southern America	Argentina	2010	10179	V_E
KC692516.1	Southern America	Argentina	2010	10179	V_E
KC692517.1	Southern America	Argentina	2010	10179	V_F
KF955427.1	Central America	Mexico	2007	10179	V_C
KF955441.1	Southern America	Venezuela	2007	10165	V_E
KF955442.1	Central America	Mexico	2007	10179	V_C
KF955443.1	Central America	Mexico	2007	10179	V_C
KF973454.1	Central America	Nicaragua	2012	10179	V_B
KF973455.1	Central America	Nicaragua	2012	10179	V_B
KF973457.1	Central America	Nicaragua	2012	10179	V_C
KF973460.1	Central America	Nicaragua	2011	10179	V_B
KF973463.1	Central America	Nicaragua	2012	10179	V_B
KF973464.1	Central America	Nicaragua	2012	10179	V_C
KF973466.1	Central America	Nicaragua	2012	10179	V_B
KF973468.1	Central America	Nicaragua	2012	10179	V_C
KF973469.1	Central America	Nicaragua	2012	10179	V_C
KF973470.1	Central America	Nicaragua	2012	10179	V_C
KF973471.1	Central America	Nicaragua	2012	10179	V_C
KF973472.1	Central America	Nicaragua	2012	10179	V_B
KF973474.1	Central America	Nicaragua	2012	10179	V_B
KF973475.1	Central America	Nicaragua	2012	10179	V_B
KJ189302.1	Southern America	Colombia	1998	10179	V
KJ189303.1	Southern America	Colombia	1998	10179	V_G

KJ189304.1	Southern America	Colombia	2005	10179	V_G
KJ189351.1	Caribbean	Puerto Rico	2012	10179	V_E
KJ189352.1	Caribbean	Puerto Rico	2012	10179	V_E
KJ189356.1	Caribbean	Puerto Rico	2012	10179	V_E
KJ189357.1	Caribbean	Puerto Rico	2012	10179	V_E
KJ189358.1	Caribbean	Puerto Rico	2012	10179	V_E
KJ189359.1	Caribbean	Puerto Rico	2012	10179	V_E
KP188540.1	Southern America	Brazil	2011	10179	V_E
KP188541.1	Southern America	Brazil	2011	10179	V_E
KP188542.1	Southern America	Brazil	2011	10179	V_E
KT279761.2	Caribbean	Haiti	2014	10179	V_E
KU509249.1	Caribbean	Jamaica	2012	10179	V_E
KU509252.1	Southern America	Venezuela	2010	10179	V_E
KU509254.1	Southern America	Venezuela	2011	10179	V_F
KY474303.1	Southern America	Ecuador	2014	10179	V_D
KY474305.1	Southern America	Ecuador	2014	10179	V_F
KY474306.1	Southern America	Ecuador	2014	10179	V_F
KY474307.1	Southern America	Ecuador	2014	10179	V_D
MF004384.1	Europe	France	2014	10179	V_B
MF797878.1	Southern America	Ecuador	2014	10179	V_D
MG877553.1	Caribbean	Haiti	2012	10179	V_E
MH450297.1	Southern America	Venezuela	2015	10179	V_D
MH450301.2	Southern America	Venezuela	2015	10179	V_D
MH450303.1	Southern America	Venezuela	2015	10179	V_D
MH450304.1	Southern America	Venezuela	2010	10179	V_D
MH450306.1	Southern America	Venezuela	2010	10179	V_F
MH450312.1	Southern America	Venezuela	2015	10179	V_D
MN449007.1	Southern America	Ecuador	2015	10176	V_D
MN449008.1	Southern America	Ecuador	2015	10176	V_D
MN449009.1	Southern America	Ecuador	2015	10176	V_D
MN449010.1	Southern America	Ecuador	2015	10176	V_D
MN449011.1	Southern America	Ecuador	2015	10176	V_D
MN449012.1	Southern America	Ecuador	2015	10176	V_D
MN449013.1	Southern America	Ecuador	2014	10176	V_D
MN449014.1	Southern America	Ecuador	2015	10176	V_D
MN449015.1	Southern America	Ecuador	2015	10176	V_D
MN449016.1	Southern America	Ecuador	2015	10176	V_D
MN556095.1	Southern America	Ecuador	2017	10179	V_D
MosquitoPool-ITA	Europe	Italy	2023	10152	V_D
MT899083.1	Central America	Mexico	2019	10179	V_D
MZ008439.1	Central America	Nicaragua	2013	10179	V_B
MZ008441.1	Central America	Nicaragua	2013	10179	V_C
MZ008442.1	Central America	Nicaragua	2013	10179	V_B
MZ008443.1	Central America	Nicaragua	2013	10179	V_B
MZ008444.1	Central America	Nicaragua	2013	10179	V_C
MZ008445.1	Central America	Nicaragua	2013	10179	V_B
MZ008446.1	Central America	Nicaragua	2013	10179	V_C
MZ008447.1	Central America	Nicaragua	2013	10179	V_B
MZ008448.1	Central America	Nicaragua	2013	10179	V_B
MZ008449.1	Central America	Nicaragua	2013	10179	V_B
MZ008451.1	Central America	Nicaragua	2013	10179	V_B
MZ008453.1	Central America	Nicaragua	2013	10179	V_C
MZ773407.1	Southern America	Colombia	2019	10179	V_D

MZ945530.1	Northern America	Texas	2020	10179	V_D
OK605754.1	Southern America	Brazil	1997	10179	V
OK605756.1	Southern America	Ecuador	2007	10179	V_E
OM654347.1	Southern America	Colombia	2021	10179	V_D
OM654348.1	Southern America	Colombia	2021	10179	V_D
OM909246.1	Northern America	Florida	2020	10179	V_E
ON123582.1	Southern America	Perù	2021	10179	V_D
ON123583.1	Southern America	Perù	2021	10179	V_D
ON123584.1	Southern America	Perù	2021	10179	V_D
ON123585.1	Southern America	Perù	2021	10179	V_D
ON123586.1	Southern America	Perù	2021	10179	V_D
ON123587.1	Southern America	Perù	2021	10179	V_D
ON123589.1	Southern America	Perù	2021	10179	V_D
ON123590.1	Southern America	Perù	2021	10179	V_D
ON123591.1	Southern America	Perù	2021	10179	V_D
ON123592.1	Southern America	Perù	2021	10179	V_D
ON123593.1	Southern America	Perù	2021	10179	V_D
ON123594.1	Southern America	Perù	2021	10179	V_D
ON123595.1	Southern America	Perù	2021	10179	V_D
ON123596.1	Southern America	Perù	2021	10179	V_D
ON123597.1	Southern America	Perù	2021	10179	V_D
ON123598.1	Southern America	Perù	2021	10179	V_D
ON123599.1	Southern America	Perù	2021	10179	V_D
ON123600.1	Southern America	Perù	2021	10179	V_D
ON123601.1	Southern America	Perù	2021	10179	V_D
ON123602.1	Southern America	Perù	2021	10179	V_D
ON123603.1	Southern America	Perù	2021	10179	V_D
ON123604.1	Southern America	Perù	2021	10179	V_D
ON426300.1	Southern America	Brazil	2021	10179	V_D
ON426317.1	Southern America	Brazil	2021	10179	V_D
ON426342.1	Southern America	Brazil	2022	10179	V_D
ON632040.1	Southern America	Brazil	2022	10179	V_D
ON632060.1	Southern America	Brazil	2022	10179	V_D
ON632069.1	Southern America	Brazil	2022	10179	V_D
ON632110.1	Southern America	Brazil	2022	10179	V_D
OP895911.1	Caribbean	Saint Martin	2021	10179	V_E
OP895912.1	Caribbean	Saint Martin	2021	10179	V_E
OQ445881.1	Northern America	Florida	2022	10176	V_D
OQ567805.1	Southern America	Paraguay	2022	10158	V_D
OQ567815.1	Southern America	Paraguay	2022	10123	V_D
OQ567818.1	Southern America	Paraguay	2022	10136	V_D
OQ567819.1	Southern America	Paraguay	2022	10010	V_D
OQ603259.1	Southern America	Colombia	2013	10179	V_D
OQ603261.1	Southern America	Colombia	2015	10179	V_D
OQ603262.1	Southern America	Colombia	2016	10179	V_D
OQ603263.1	Southern America	Colombia	2016	10179	V_D
OQ786010.1	Southern America	Brazil	2023	10179	V_D
OQ786036.1	Southern America	Brazil	2023	10179	V_D
OQ821333.1	Caribbean	Cuba	2019	10179	V_D
OQ821336.1	Caribbean	Cuba	2019	10179	V_D
OQ821338.1	Caribbean	Cuba	2020	10179	V_D
OQ821339.1	Caribbean	Cuba	2020	10179	V_D
OQ821347.1	Caribbean	Cuba	2022	10179	V_D

OQ821349.1	Caribbean	Cuba	2022	10179	V_D
OQ821351.1	Caribbean	Cuba	2022	10179	V_D
OQ821358.1	Caribbean	Dominican Republic	2019	10179	V_E
OQ821371.1	Northern America	Florida	2010	10179	V_C
OR025596.1	Southern America	Brazil	2023	10179	V_D
OR025598.1	Southern America	Brazil	2023	10179	V_D
OR025629.1	Southern America	Brazil	2023	10179	V_D
OR025634.1	Southern America	Brazil	2023	10179	V_D
OR025643.1	Southern America	Brazil	2023	10179	V_D
OR025645.1	Southern America	Brazil	2023	10179	V_D
OR025653.1	Southern America	Brazil	2023	10179	V_D
OR167359.1	Southern America	Brazil	2023	10036	V_D
OR167364.1	Southern America	Brazil	2023	10025	V_D
OR167366.1	Southern America	Brazil	2023	10135	V_D
OR167377.1	Southern America	Brazil	2023	10013	V_D
OR167378.1	Southern America	Brazil	2023	10096	V_D
OR229954.1	Caribbean	Guadeloupe	2020	10179	V_E
OR229955.1	Caribbean	Guadeloupe	2020	10179	V_E
OR229956.1	Caribbean	Guadeloupe	2020	10179	V_E
OR229957.1	Caribbean	Guadeloupe	2020	10179	V_D
OR258403.1	Southern America	Brazil	2022	10115	V_D
OR258406.1	Southern America	Brazil	2022	10125	V_D
OR258408.1	Southern America	Brazil	2022	10023	V_D
OR258411.1	Southern America	Brazil	2022	10025	V_D
OR258415.1	Southern America	Brazil	2022	10028	V_D
OR258416.1	Southern America	Brazil	2022	10047	V_D
OR258417.1	Southern America	Brazil	2022	10031	V_D
OR258419.1	Southern America	Brazil	2022	10133	V_D
OR258421.1	Southern America	Brazil	2022	10089	V_D
OR258422.1	Southern America	Brazil	2022	10021	V_D
OR258423.1	Southern America	Brazil	2022	10051	V_D
OR258428.1	Southern America	Brazil	2022	10134	V_D
OR258429.1	Southern America	Brazil	2022	10114	V_D
OR258430.1	Southern America	Brazil	2022	10152	V_D
OR258431.1	Southern America	Brazil	2022	10043	V_D
OR258434.1	Southern America	Brazil	2022	10126	V_D
OR258435.1	Southern America	Brazil	2022	10150	V_D
OR258436.1	Southern America	Brazil	2022	10132	V_D
OR258439.1	Southern America	Brazil	2022	10057	V_D
OR258441.1	Southern America	Brazil	2022	10021	V_D
OR258442.1	Southern America	Brazil	2022	10021	V_D
OR258443.1	Southern America	Brazil	2022	10114	V_D
OR258447.1	Southern America	Brazil	2022	10101	V_D
OR258448.1	Southern America	Brazil	2022	10114	V_D
OR258452.1	Southern America	Brazil	2022	10114	V_D
OR258453.1	Southern America	Brazil	2022	10027	V_D
OR258457.1	Southern America	Brazil	2022	10023	V_D
OR258458.1	Southern America	Brazil	2022	10021	V_D
OR258459.1	Southern America	Brazil	2022	10021	V_D
OR258461.1	Southern America	Brazil	2022	10035	V_D
OR258463.1	Southern America	Brazil	2022	10044	V_D
OR258465.1	Southern America	Brazil	2022	10025	V_D
OR258466.1	Southern America	Brazil	2022	10108	V_D

OR258468.1	Southern America	Brazil	2022	10021	V_D
OR258469.1	Southern America	Brazil	2022	10089	V_D
OR258470.1	Southern America	Brazil	2022	10102	V_D
OR258471.1	Southern America	Brazil	2022	10021	V_D
OR258472.1	Southern America	Brazil	2022	10021	V_D
OR258473.1	Southern America	Brazil	2022	10021	V_D
OR258474.1	Southern America	Brazil	2022	10021	V_D
OR258475.1	Southern America	Brazil	2022	10114	V_D
OR258478.1	Southern America	Brazil	2022	10021	V_D
OR258482.1	Southern America	Brazil	2022	10114	V_D
OR258483.1	Southern America	Brazil	2022	10021	V_D
OR258486.1	Southern America	Brazil	2022	10100	V_D
OR258488.1	Southern America	Brazil	2022	10043	V_D
OR258489.1	Southern America	Brazil	2022	10114	V_D
OR258490.1	Southern America	Brazil	2022	10069	V_D
OR258491.1	Southern America	Brazil	2022	10021	V_D
OR258492.1	Southern America	Brazil	2022	10021	V_D
OR258493.1	Southern America	Brazil	2022	10021	V_D
OR258497.1	Southern America	Brazil	2022	10119	V_D
OR258500.1	Southern America	Brazil	2022	10075	V_D
OR258501.1	Southern America	Brazil	2022	10021	V_D
OR258502.1	Southern America	Brazil	2022	10133	V_D
OR258504.1	Southern America	Brazil	2022	10026	V_D
OR258505.1	Southern America	Brazil	2022	10120	V_D
OR258506.1	Southern America	Brazil	2022	10125	V_D
OR258507.1	Southern America	Brazil	2022	10136	V_D
OR258512.1	Southern America	Brazil	2022	10021	V_D
OR258513.1	Southern America	Brazil	2022	10114	V_D
OR258514.1	Southern America	Brazil	2022	10021	V_D
OR258516.1	Southern America	Brazil	2022	10025	V_D
OR258517.1	Southern America	Brazil	2022	10114	V_D
OR258518.1	Southern America	Brazil	2022	10099	V_D
OR258519.1	Southern America	Brazil	2022	10114	V_D
OR258522.1	Southern America	Brazil	2022	10114	V_D
OR258524.1	Southern America	Brazil	2022	10140	V_D
OR258529.1	Southern America	Brazil	2022	10114	V_D
OR258530.1	Southern America	Brazil	2022	10114	V_D
OR258531.1	Southern America	Brazil	2022	10143	V_D
OR258533.1	Southern America	Brazil	2022	10114	V_D
OR258534.1	Southern America	Brazil	2022	10021	V_D
OR258535.1	Southern America	Brazil	2022	10021	V_D
OR258537.1	Southern America	Brazil	2022	10100	V_D
OR258547.1	Southern America	Brazil	2022	10107	V_D
OR258548.1	Southern America	Brazil	2022	10114	V_D
OR258549.1	Southern America	Brazil	2022	10021	V_D
OR258550.1	Southern America	Brazil	2022	10124	V_D
OR258551.1	Southern America	Brazil	2022	10025	V_D
OR258552.1	Southern America	Brazil	2022	10093	V_D
OR258563.1	Southern America	Brazil	2022	10129	V_D
OR258564.1	Southern America	Brazil	2022	10094	V_D
OR258565.1	Southern America	Brazil	2022	10102	V_D
OR258571.1	Southern America	Brazil	2022	10034	V_D
OR258572.1	Southern America	Brazil	2022	10114	V_D

OR389009.1	Southern America	Colombia	2004	10179	V_G
OR389012.1	Southern America	Perù	2000	10179	V
OR389014.1	Caribbean	Puerto Rico	2006	10179	V_E
OR389272.1	Caribbean	Bahamas	1977	10179	V
OR389273.1	Southern America	Brazil	1986	10179	V
OR389274.1	Southern America	Brazil	1986	10179	V
OR389276.1	Southern America	Colombia	1990	10179	V
OR389277.1	Southern America	Ecuador	1988	10179	V
OR389292.1	Caribbean	Jamaica	1977	10179	V
OR389303.1	Southern America	Trinidad and Tobag	1978	10179	V
OR389304.1	Southern America	Trinidad and Tobag	1978	10179	V
OR518216.1	Southern America	Brazil	2021	10179	V_E
OR518217.1	Southern America	Brazil	2021	10179	V_F
OR518218.1	Southern America	Brazil	2021	10179	V_F
OR518223.1	Southern America	Brazil	2021	10179	V_F
OR518224.1	Southern America	Brazil	2021	10179	V_E
OR518225.1	Southern America	Brazil	2021	10179	V_F
OR518226.1	Southern America	Brazil	2021	10179	V_E
OR518234.1	Southern America	Brazil	2021	10179	V_E
OR518236.1	Southern America	Brazil	2021	10179	V_E
OR518242.1	Southern America	Brazil	2021	10179	V_D
OR518245.1	Southern America	Brazil	2021	10179	V_E
OR518251.1	Southern America	Brazil	2021	10179	V_E
OR518252.1	Southern America	Brazil	2021	10179	V_D
OR518256.1	Southern America	Brazil	2021	10179	V_E
OR518266.1	Southern America	Brazil	2022	10179	V_E
OR654272.1	Northern America	Florida	2023	10179	V_D
OR654273.1	Northern America	Florida	2023	10179	V_D
OR654282.1	Northern America	Florida	2023	10179	V_D
OR771117.1	Northern America	Florida	2023	10179	V_E
OR771122.1	Northern America	Florida	2023	10179	V_E
PP234945.1	Caribbean	Dominican Republic	2019	10179	V_E
PP234962.1	Southern America	Colombia	2018	10179	V_D
